# Botulinum Neurotoxin A Complex Recognizes Host Carbohydrates through Its Hemagglutinin Component

**DOI:** 10.3390/toxins6020624

**Published:** 2014-02-12

**Authors:** Guorui Yao, Kwangkook Lee, Shenyan Gu, Kwok-Ho Lam, Rongsheng Jin

**Affiliations:** Department of Physiology and Biophysics, University of California, Irvine, CA 92697, USA; E-Mails: g.yao@uci.edu (G.Y.); kwanglee@uci.edu (K.L.); sgu5@its.jnj.com (S.G.); kwokhl@uci.edu (K.H.L.)

**Keywords:** Botulinum neurotoxins (BoNTs), progenitor toxin complex (PTC), hemagglutinin (HA), carbohydrate receptor, glycan array

## Abstract

Botulinum neurotoxins (BoNTs) are potent bacterial toxins. The high oral toxicity of BoNTs is largely attributed to the progenitor toxin complex (PTC), which is assembled from BoNT and nontoxic neurotoxin-associated proteins (NAPs) that are produced together with BoNT in bacteria. Here, we performed *ex vivo* studies to examine binding of the highly homogeneous recombinant NAPs to mouse small intestine. We also carried out the first comprehensive glycan array screening with the hemagglutinin (HA) component of NAPs. Our data confirmed that intestinal binding of the PTC is partly mediated by the HA moiety through multivalent interactions between HA and host carbohydrates. The specific HA-carbohydrate recognition could be inhibited by receptor-mimicking saccharides.

## 1. Introduction

Botulinum neurotoxins (BoNTs) are among the most life threatening natural substances to man, which have been designated as the Tier 1 select agents by the Centers for Disease Control and Prevention (CDC) [1,2]. Paradoxically, BoNTs are also powerful medicines and popular cosmetics. In foodborne botulism, which is caused by eating BoNT-contaminated foods, BoNTs are absorbed from the gastrointestinal (GI) tract to the general circulation. They invade motoneurons at neuromuscular junctions and cleave the soluble *N*-ethylmaleimide sensitive factor attachment protein receptors (SNAREs) complex, which subsequently inhibits neurotransmitter release and paralyzes the affected muscles [3,4,5]. The current treatment for oral BoNT intoxication relies on early diagnosis and immediate administration of an equine antitoxin [2]. It is an urgent need to understand how BoNTs are absorbed in the GI tract, which will help develop preventive countermeasures against BoNTs that stop toxin invasion in the first place.

There are eight major serotypes (termed A–H) of BoNTs [6,7], which are produced in bacteria together with non-toxic neurotoxin-associated proteins (NAPs) in the form of progenitor toxin complex (PTC) [8]. BoNT/A, B, C, D and G are naturally produced together with four NAPs including non-toxic non-hemagglutinin (NTNHA) and three hemagglutinins (HAs: HA33, HA17 and HA70, termed HA1, HA2 and HA3, respectively) [8]. However, some other serotypes, such as BoNT/E and F, do not have the *ha* genes, but contain genes of unknown function (terms *orfX*) [6,9]. We focus our studies on BoNT/A because it is the major cause of human botulism and also the most common BoNT for clinical uses.

It has been reported that BoNT/A alone may be sufficient to penetrate the GI barrier by transcytosis, which could be mediated by the C-terminal receptor-binding domain of BoNT/A [10,11,12]. Remarkably, the oral toxicity of the large PTC (L-PTC, also termed the 16S complex) of BoNT/A is increased by hundreds of folds in comparison to that of the free toxin [13,14,15]. It thus strongly suggests that NAPs play a critical role during oral ingestion of BoNT/A. We have previously shown that BoNT/A and NTNHA form an interlocked minimally functional complex (M-PTC/12S complex), in which BoNT is protected by NTNHA against digestive proteases and the acidic environment of the GI tract [16]. However, the functional role of the HA proteins is not fully understood. Based on recently reported structures of a 12-subunit protein complex composed of HA70, HA17, and HA33 (termed the HA complex) [17,18], it is clear that the HA complex exhibits multiple carbohydrate-binding sites located on HA70 and HA33. Previous *in vitro* biophysical and biochemical studies suggested that the HA complex enhances intestinal absorption of the toxin complex by enriching PTCs on the cell surface through multivalent binding with carbohydrates [17]. Here, we conducted *ex vivo* binding assays to further examine binding of the different BoNT/A PTC components to mouse small intestine tissues. Furthermore, we performed the first comprehensive functional glycomics screening to address the carbohydrate-binding specificity of the HA proteins.

## 2. Results and Discussions

### 2.1. Homogeneous Recombinant NAPs Are Unique Molecular Probes for Functional Studies

**Figure 1:**
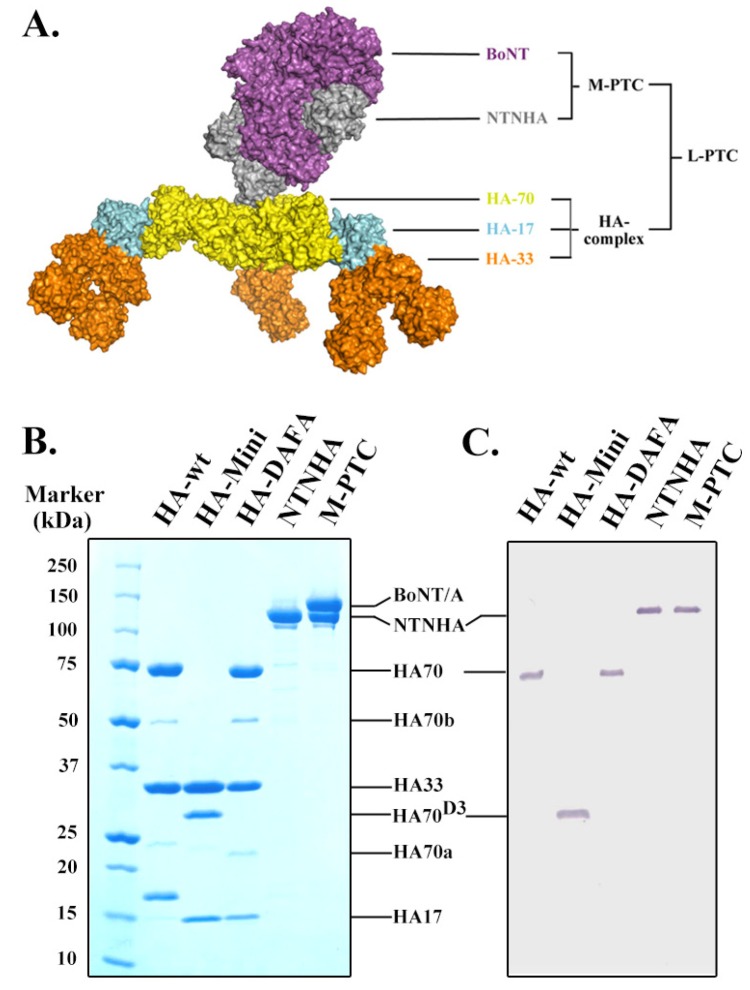
Architecture of the large progenitor toxin complex (L-PTC) (16S complex). (**A**) Surface representation of the L-PTC of botulinum neurotoxin (BoNT/A) [17]. The M-PTC (12S complex) is composed of BoNT/A (magenta) and NTNHA (gray). The three-blade shaped hemagglutinin (HA) complex comprises three HA70 (HA3, yellow), three HA17 (HA2, cyan), and six HA33 (HA1, orange). (**B**) A SDS-PAGE showing the purity of the various recombinant PTC components. Please note that a small amount of HA70 was spontaneously nicked into two peptides (HA70a and HA70b) as previously reported [17]; HA70 and HA17 in the HA-wt complex had uncleaved His-tags. (**C**) A Western blotting using the anti-myc antibody confirmed the integrity of the myc-tag on HA70 (lanes 1, 3), HA70^D3^ (lane 2), and NTNHA (lanes 4, 5).

Many earlier researches on NAPs relied on samples purified from natural sources. Sometimes, the individual HA protein had to be stripped from PTC with harsh conditions. It thus raises the concern of contamination with other clostridial proteins. In this regard, we have developed a robust system to overexpress recombinant NTNHA and all three HAs in *E. coli* and purify them to high homogeneity [17]. The high quality of these proteins has been demonstrated by their successful crystallization [16,17]. The three HAs assemble into a three-fold symmetric complex (HA-wt) that exhibits three identical blades (Figure 1A) [17]. The central hub of the HA complex is composed of the homo-trimeric D1–2 domain of HA70 (residues Met1–Asp377) while each blade (termed HA-mini) is composed of the D3 domain of HA70 (HA70^D3^, residues Pro378–Asn626), one molecule of HA17, and two HA33 molecules. It is worth noting that the carbohydrate-binding site on HA70 is located on the D3 domain. Therefore, the HA-mini complex maintains the intact carbohydrate-binding sites on both HA70 and HA33, except that it is monomeric as opposed to the trimeric HA complex.

To systematically investigate the PTC–carbohydrate interactions, we reconstituted the complete HA-wt and the HA-mini complexes. In addition, we produced a carbohydrate-binding deficient HA complex, which carries two mutations (D263A/F278A) on the galactose-binding site on HA33 (termed HA-DAFA) [17]. The recombinant NTNHA and the M-PTC (composed of NTNHA and BoNT/Ai, a catalytically inactive BoNT/A) were also produced as previously described [16]. All protein samples were purified to high homogeneity using a combination of nickel affinity chromatography, ion-exchange chromatography, and gel filtration (Figure 1B). Furthermore, a myc-tag was introduced to the C-terminus of HA70 and HA70^D3^, or to the *N*-terminus of NTNHA, to allow immunofluorescence-based analysis. The integrity of the myc-tag on the final purified protein samples was confirmed by a Western blotting (Figure 1C).

### 2.2. The HA Complex Mediates the Intestinal Binding of the PTC

Although BoNTs can be absorbed in many areas of the alimentary canal including the stomach, small intestine, and large intestine [19,20], the upper part of the small intestine is considered the most important site for BoNT absorption [14]. Therefore, we conducted *ex vivo* binding assays using freshly prepared mouse jejunum segments (~8–14 cm distal to the stomach). Briefly, the jejunum segments were incubated with various protein samples for ~30 min at 4 °C followed by immunofluorescence images of myc-tagged HA complex using an anti-myc antibody and a secondary antibody conjugated to Alexa Fluor 594. DAPI was applied to visualize the cell nuclei.

**Figure 2:**
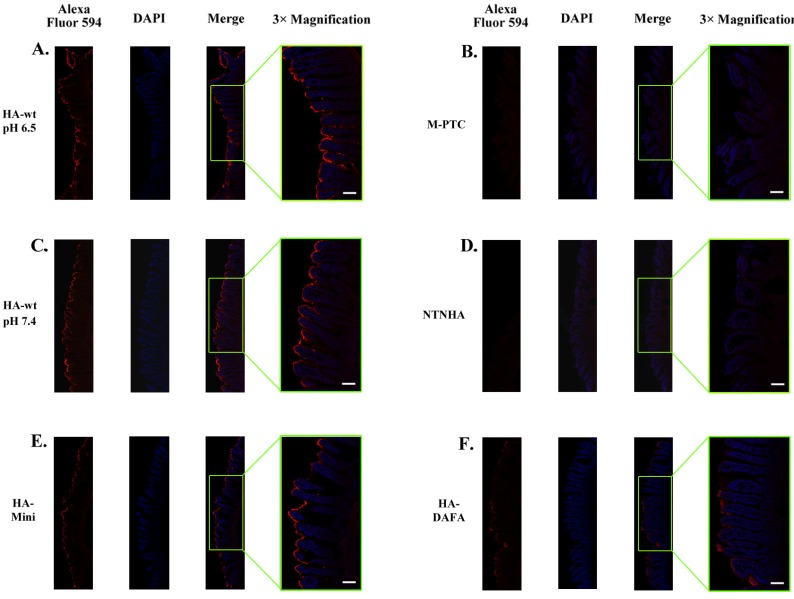
Intestinal binding of the PTC is primarily mediated by the HA complex. Same amount of the myc-tagged HA-wt, HA-mini, HA-DAFA, M-PTC, and non-toxic non-hemagglutinin (NTNHA) were incubated with mouse jejunum segments separately. The tissues were fixed, immunostained with a monoclonal anti-myc antibody, and visualized with a secondary antibody conjugated with Alexa Fluor 594 (red). The nuclei of the epithelial cells were labeled with DAPI (blue). The HA-wt complex showed a robust binding on luminal surface of the small intestine at both pH (**A**) 6.5 and (**C**) 7.4. In contrast, the (**B**) M-PTC and (**D**) NTNHA did not have detectable binding signal. (**E**) The HA-mini complex showed a similar binding pattern and intensity in comparison to the HA-wt complex. (**F**) The HA-DAFA mutant showed a significantly decreased binding. Images with 3× magnification were shown to the right of each panel. A minimum of three slides were prepared and analyzed for each sample, and the experiments were repeated using two mice. One slide of each sample was shown as representative. Scale bars: 0.2 mm.

To maintain the tissues in a physiological condition, an incubation buffer at pH 7.4 was used for most of the binding experiments except for the M-PTC. The M-PTC is known to dissemble at non-acidic pH, which is needed to release BoNT from the complex upon entering the circulation [16,21]. Therefore, binding of the M-PTC was tested at pH 6.5. We also examined the HA-wt complex at pH 6.5 as a control. A strong binding of the HA-wt complex was observed on the lumen of the small intestine with the HA complex evenly distributed on the surface of villi. Two studies performed at pH 6.5 and 7.4 showed an almost indistinguishable binding profile for the HA-wt complex (Figure 2A,C). The HA-mini complex, representing one arm of the complete complex, also showed a strong binding, although a close examination revealed a slightly less homogeneous binding on villi in comparison to the HA-wt (Figure 2E). In contrast, neither the M-PTC (Figure 2B) nor NTNHA (Figure 2D) showed detectable binding signal. Taken together, these data suggest that intestinal binding of the toxin complex largely depends on the HA complex instead of the M-PTC.

### 2.3. The Intestinal Binding of the PTC Relies on Carbohydrate Receptors

Our previous *in vitro* studies have demonstrated that HA70 and HA33 specifically recognize sialic acid and galactose, respectively [17]. Here, we performed two *ex vivo* binding assays to further probe the functional role of the HA–carbohydrate interaction under a physiological condition. First, we examined the intestinal binding of a carbohydrate-binding deficient HA complex, the HA-DAFA complex, which cannot bind galactose (Gal) [17]. When incubated with the mouse jejunum tissues, the HA-DAFA complex displayed a significantly decreased binding efficiency when compared to the HA-wt complex (Figure 2F). Thus, the loss of Gal-binding ability of HA33 directly decreased the intestinal binding efficiency of the HA complex. In line with this result, a previous study showed that the HA-DAFA complex failed to disrupt the fully differentiated Caco-2 cell monolayers [17]. These findings provide a strong support for the crucial role of the HA–carbohydrate interaction during toxin absorption.

**Figure 3:**
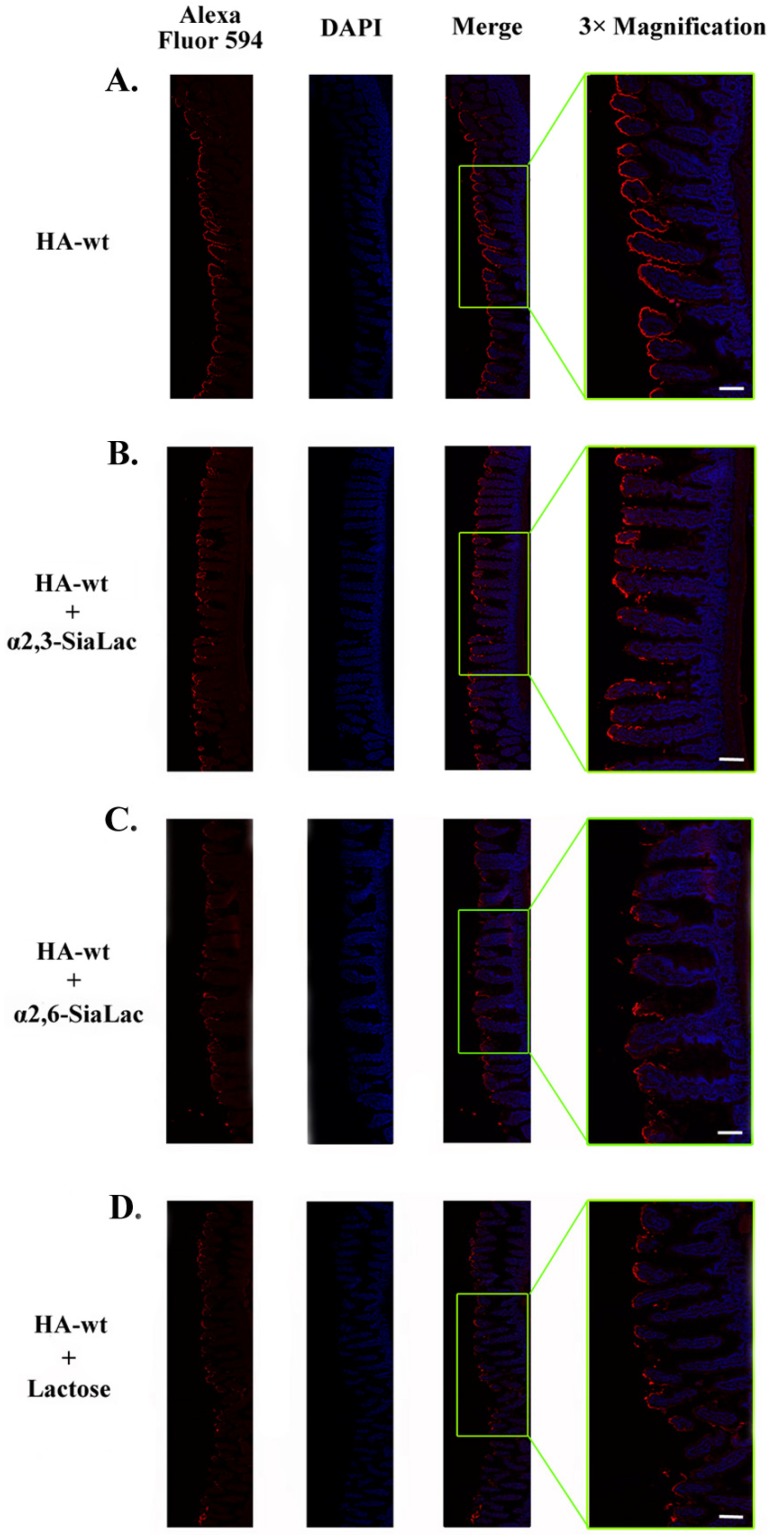
The receptor-mimicking saccharides significantly decrease the intestinal binding of the HA complex. Mouse jejunum segments were incubated with (**A**) the HA-wt complex or with the same amount of complex in the presence of (**B**) α2,3-SiaLac, (**C**) α2,6-SiaLac, or (**D**) lactose. The HA complex and the cell nuclei were visualized as described above. All three saccharides significantly inhibited binding of the HA complex to the intestinal lumen. Scale bars: 0.2 mm.

We also conducted a competitive binding assay using three receptor-mimicking saccharides, α2,3- or α2,6-sialyllactose (α2,3-, or α2,6-SiaLac) and lactose, which are known to bind HA70 and HA33, respectively. We found that, in the presence of these compounds, the binding of the HA-wt complex on the luminal surface of mouse jejunum displayed a heterogeneous pattern with decreased binding signals (Figure 3). It suggested that the receptor-mimicking saccharides competed with the endogenous intestinal carbohydrates for HA binding. Collectively, these data further supported the notion that the Gal- and sialic acid-containing carbohydrates in the small intestine are bona fide receptors for BoNT PTCs.

### 2.4. The Specific HA–Carbohydrate Recognition Primarily Depends on the Terminal Saccharides

**Figure 4:**
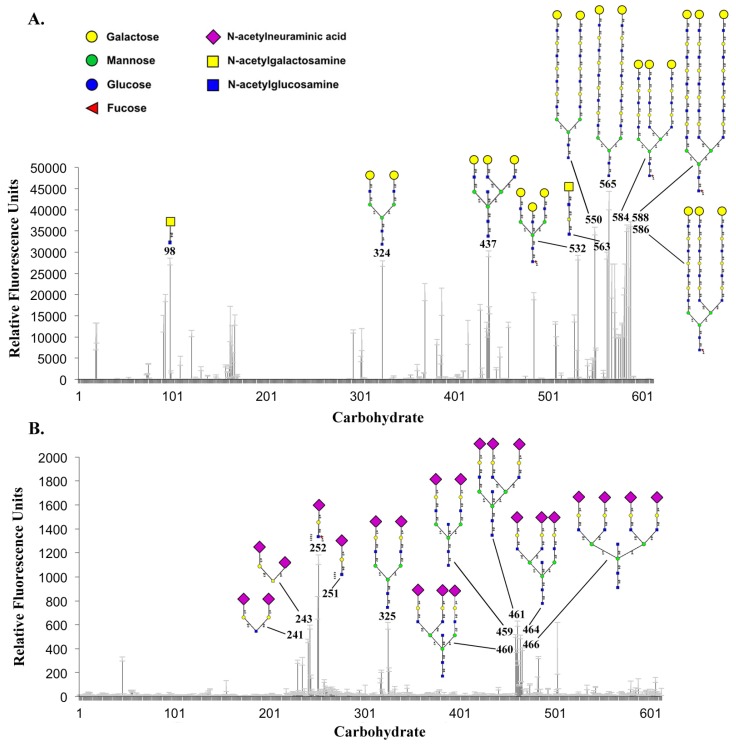
Glycan array screenings with the complete HA complex and HA70. Mammalian printed array (Version 5.1) of the Consortium for Functional Glycomics (CFG) was probed with the (**A**) HA complex (100 μg/mL) and (**B**) HA70 (1 mg/mL). Ten glycans that yielded the highest binding signals are shown with their ID numbers and structures. The HA complex mostly binds to the glycans with a terminal Gal, especially the ones with a terminal Gal-GlcNAc. HA70 mostly recognizes the terminal Neu5Ac, with a preference for Neu5Ac-Gal-GlcNAc structure. Error bars: standard deviations of four measurements.

In spite of a wealth of information on HA–carbohydrate interactions [22,23,24,25,26], the receptor-binding specificity of the HA proteins has not been systematically addressed. To fill in the knowledge gap, we performed comprehensive glycan array screenings at the Core H of the Consortium for Functional Glycomics (CFG) using the mammalian glycan array (version 5.1) that contains a broad-spectrum of natural/synthetic glycans (610 glycans in total). An Alexa Fluor^®^ 488-labeled HA70 was analyzed at 1000 and 200 μg/mL concentrations (equivalent to 4.8 and 0.96 μM). The complete HA-wt complex containing the labeled HA70 was probed at concentrations of 1000, 100, 30 and 10 μg/mL (equivalent to 2.1, 0.21, 0.07 and 0.02 μM). The screening results are of open access and can be found online (Screen IDs: primscreen_5567-5570, 5735-5738) [27]. Representative data are shown in Figure 4.

The HA-wt complex and the standalone HA70 showed very different glycan-binding preferences and sensitivities. For the HA-wt complex, the carbohydrates yielding the highest binding signals were those bearing a terminal Gal, especially the ones with a terminal Gal-GlcNAc (*N*-acetylglucosamine) structure. The HA complex also recognized, to a lesser extent, the glycans with terminal *N*-acetylgalactosamin (GalNAc) (Figure 4A). The best hits for HA70 were the glycans with a terminal *N*-acetylneuraminic acid (Neu5Ac), especially the ones possess a terminal Neu5Ac-Gal-GlcNAc structure (Figure 4B). Interestingly, HA70 and the HA complex do not have a strict preference for specific saccharides beyond the terminal Neu5Ac or Gal. Therefore, the HA complex is able to efficiently accumulate the PTC on the intestinal lumen, where the glycans with the terminal Gal or Neu5Ac are highly abundant.

We noticed that the complete HA-wt complex yielded much stronger binding signals than the free HA70 during the screening (Figure 4A,B). Although thermodynamic studies in solution suggested that the binding affinity between HA33 and galactose is similar to that between HA70 and Neu5Ac [17], the binding avidity of the HA complex to the immobilized glycans on a chip is correlated to the strength of multivalent binding. Therefore, the strong binding of the HA complex was predominantly contributed by the six Gal-binding sites on HA33. In comparison, signals of Neu5Ac binding to the three sites on HA70 were weaker, which were likely masked by the strong Gal binding on HA33 in the context of the HA complex.

## 3. Experimental Section

### 3.1. Cloning, Protein Expression, and Purification

*C. botulinum* strain 62A-derived HA70 (residues Met1–Asn626), HA70^D3^ (residues Pro378–Asn626), HA17 (residues Met1–Ile146), HA33 (residues Met1–Pro293), HA33-D263A/F278A, and NTNHA were cloned, expressed and purified as previously described [16,17]. The HA-wt, HA-mini complexes were produced by assembling the HA17–HA33 subcomplex with HA70 or HA70^D3^, respectively. The HA-DAFA complex was assembled using HA70 and the HA17–HA33^D263A/F278A^ subcomplex. The genetically modified, catalytically inactive BoNT/Ai that carries three mutations (E224Q/R363A/Y366F) in the catalytic site was a generous gift from Dr. Andreas Rummel (Institut für Toxikologie, Medizinische Hochschule Hannover, Hannover, Germany). The M-PTC composed of BoNT/Ai and NTNHA was prepared as previously described [16].

### 3.2. Immunoblotting Assay

The purified proteins were separated by a SDS-PAGE, transferred to a nitrocellulose membrane (Bio-Rad, Hercules, CA, USA), and immunoblotted with a mouse anti-myc antibody (Cell Signaling, Danvers, MA, USA). The blot was visualized with a rabbit anti-mouse secondary antibody conjugated to alkaline phosphatase (Bio-Rad).

### 3.3. Ex Vivo Intestinal Binding Assay

Four-month-old wild-type male C57BL/6 mice were fasted for 24 h (but allowed access to water) prior to euthanasia by isoflurane inhalation. A 6 cm long jejunum (from ~8–14 cm distal to the stomach) was quickly dissected out, gently flushed once with ice-cold phosphate buffered saline (PBS) (137 mM NaCl, 2.7 mM KCl, 8 mM Na_2_HPO_4_, 1.46 mM KH_2_PO_4_, pH 7.4) and placed into the ice-cold PBS immediately. The jejunum was opened with an incision along its cephalocaudal axis, and further cut into 5 mm segments parallel to the crypt-villus axis. The segments were then incubated in 0.5 mL PBS (pH 7.4) with: (1) HA-wt; (2) HA-wt + α2,3-sialyllactose (α2,3-SiaLac); (3) HA-wt + α2,6-sialyllactose (α2,6-SiaLac); (4) HA-wt + lactose; (5) HA-mini; (6) HA-DAFA; and (7) NTNHA, respectively. The binding assay was also performed at pH 6.5 for the M-PTC and the HA-wt complex. For all of the binding assays, the protein concentration applied was 120 nM and the saccharide concentration was 40 mM. After 30 min incubation at 4 °C, the tissues were rinsed twice with PBS (pH 7.4 or 6.5), fixed with Carnoy’s solution for 1 h, washed two more times with PBS (pH 7.4), and placed in 30% sucrose/PBS solution overnight.

### 3.4. Tissue Preparation and Immunostaining

The fixed tissues were embedded in optimum cutting temperature (O.C.T.) compound in a cryo-mold placed in a 2-methylbutane bath that was precooled by dry ice. After embedding, the samples were stored at −70 °C. Before section, the tissues were placed in the cryostat for 1 h to warm up to −20 °C. Frozen sections were rinsed three times with PBS (pH 7.4), and incubated with primary antibody (anti-myc tag antibody, Abcam, Cambridge, UK) at room temperature for 1 h. After washing with three changes of PBS, the secondary antibody (Alexa Fluor anti Rabbit IgG 594, Invitrogen, Carlsbad, CA, USA) was applied and incubated with the sections for 30 min at room temperature. Samples were washed three times with PBS and then coverslipped with VectaShield Hardset Mounting Medium with DAPI (Vector Labs, Burlingame, CA, USA). All slides were scanned at a magnification of 20× using the Aperio Scanscope FL system (Aperio Technologies, Vista, CA, USA). The appropriate dyes were assigned and illumination levels were calibrated using a preset procedure, the parameters were saved and applied to all slides.

### 3.5. Glycan Array Screening

The purified HA70 was labeled with Alexa Fluor^®^ 488 carboxylic acid, succinimidyl ester (Life Technologies, Carlsbad, CA, USA) according to the manufacturer’s instruction. The labeled HA complex was prepared using the labeled HA70 and the unlabeled HA17–HA33 complex. Before screening, the fluorescently labeled samples were diluted to various concentrations in standard binding buffer (20 mM Tris-HCl, pH 7.4, 150 mM NaCl, 2 mM CaCl_2_, 2 mM MgCl_2_, 1% BSA, and 0.05% Tween 20). The screening was performed at the Core H of the CFG using the mammalian glycan array (version 5.1) [28].

## 4. Conclusions

In summary, we performed the first comprehensive glycan array screening using highly homogeneous recombinant HAs of BoNT/A. Our results clearly showed that the HA complex specifically recognizes glycans with a terminal Gal or Neu5Ac, but has no stringent preference for the subsequent saccharides. It thus enables the PTC to bind to a large variety of glycans in the intestine lumen to ensure an efficient binding. The physiological role of HA–carbohydrate interactions during intestinal absorption was confirmed by *ex vivo* binding assays using mouse small intestine tissues. Furthermore, we showed that carbohydrate recognition of the HA complex could be inhibited by receptor-mimicking saccharides. Therefore, such compounds have the potential to become preventive countermeasures for BoNTs upon pre-treatment. We speculate that such inhibitory compounds could be administered orally and take effect in the GI tract without entering the body.
